# Design and Demonstration of High-Efficiency Quantum Well Solar Cells Employing Thin Strained Superlattices

**DOI:** 10.1038/s41598-019-50321-x

**Published:** 2019-09-27

**Authors:** Roger E. Welser, Stephen J. Polly, Mitsul Kacharia, Anastasiia Fedorenko, Ashok K. Sood, Seth M. Hubbard

**Affiliations:** 1 Magnolia Optical Technologies, Inc., 52-B Cummings Park, Suite 314, Woburn, MA 01801 USA; 2 NanoPower Research Laboratories, Rochester Institute of Technology, Rochester, NY 14623 USA

**Keywords:** Solar energy and photovoltaic technology, Electrical and electronic engineering, Solar cells, Electronics, photonics and device physics

## Abstract

Nanostructured quantum well and quantum dot III–V solar cells provide a pathway to implement advanced single-junction photovoltaic device designs that can capture energy typically lost in traditional solar cells. To realize such high-efficiency single-junction devices, nanostructured device designs must be developed that maximize the open circuit voltage by minimizing both non-radiative and radiative components of the diode dark current. In this work, a study of the impact of barrier thickness in strained multiple quantum well solar cell structures suggests that apparent radiative efficiency is suppressed, and the collection efficiency is enhanced, at a quantum well barrier thickness of 4 nm or less. The observed changes in measured infrared external quantum efficiency and relative luminescence intensity in these thin barrier structures is attributed to increased wavefunction coupling and enhanced carrier transport across the quantum well region typically associated with the formation of a superlattice under a built-in field. In describing these effects, a high efficiency (>26% AM1.5) single-junction quantum well solar cell is demonstrated in a device structure employing both a strained superlattice and a heterojunction emitter.

## Introduction

Over the past three decades, a number of high power conversion efficiency solar cell concepts have been proposed that mix narrow and wide bandgap material within a single P-N junction nanostructured solar cell or as a subcell in a multijunction approach^1–3^. As a practical application, nanostructured solar cells can boost the current output of III–V multijunction structures when quantum wells or quantum dots are added to the current limiting subcell^4–6^. Nanostructured absorbers have also been associated with improved radiation resistance^7–9^. In the long term, the real value of quantum nanostructured solar cells is their promise to break traditional limits on photovoltaic performance^10^. In particular, III–V nanostructures have been investigated as a means of implementing advanced device designs that leverage processes employing optical up-conversion via an intermediate band^11,12^, hot carrier extraction^13,14^, and/or restricted luminescent emissions^15^. All of these advanced device concepts require, in one way or another, a reduction in non-radiative recombination throughout the device and the inhibition of radiative recombination within the nanostructured materials. Detailed balance calculations suggest that in theory, single-junction devices could achieve an efficiency of 40% or more with any of these advanced device concepts^10^. In this work, wide energy gap emitter and window layers are employed to help minimize non-radiative recombination, while the use of thin barrier quantum well superlattice structures shows signs of reduced radiative recombination.

The addition of lower energy gap material, including quantum wells (QW) and quantum dots, to the GaAs absorber region provides a clear pathway to further enhance performance by extending infrared absorption. To achieve these lower bandgaps, alternate binary or ternary materials can be used, but require strained, metamorphic growth due to differences in lattice constant between the bulk absorber and the nanostructure. In practice, device efficiency will depend on the precise impact of the nanostructured layers on the dark current and the overall collection efficiency^16^. Demonstrating high-efficiency single-junction QW solar cell devices has proven challenging^17^. Past work on QW solar cells has often focused on increasing the sub-bandgap collection, and therefore the short circuit current density (J_SC_), by employing strain-compensation techniques to add as many layers as possible to the QW absorber region^18^. Strain compensation techniques attempt to avoid the formation of strain-induced defects in lattice mismatched material systems by balancing, rather than simply minimizing, strain buildup. However, the one-sun efficiency of the best reported strain-balanced QW devices have been limited to less than 25% by sub-optimal open circuit voltage (V_OC_) values, typically less than 1.0 V in GaAs-based cells^18,19^.

Prior work on strained QW solar cells has suggested that the emitter layer and QW region designs can be optimized to reduce both the radiative and non-radiative recombination^20,21^. In addition, reports by Sugiyama *et al*. have shown that thin barrier, i.e. superlattice, quantum well solar cells lead to improved carrier collection efficiency and recovery in V_oc_^22^. In this work, the impact of inter-QW GaAs barrier thickness on solar cell absorption and emissions is systematically investigated in both N-on-P GaAs homojunction and P-on-N In_0.49_Ga_0.51_P/GaAs heterojunction device structures incorporating strained, defect-free In_0.08_Ga_0.92_As (hereafter, ‘InGaAs’) QWs. Measurements of the spectral response (SR) and luminescence characteristics of these devices indicate that reducing the GaAs barrier thickness both enhances carrier collection from the QWs and reduces radiative recombination within the strained InGaAs well region. This observation is consistent with increased carrier delocalization and separation as the barrier thickness decreases and superlattice effects dominate (e.g. miniband formation, Wannier-Stark hopping, and/or sequential tunneling)^23–26^. To further quantify the impact of barrier thickness on collection and emission from the QW region, reciprocity relations are leveraged to define an apparent radiative efficiency factor using measured external quantum efficiency (EQE) and electroluminescence (EL) spectra.

Solar cell device results are shown from both homojunction and heterojunction QW enhanced designs with thin inter-well barriers. The heterojunction design was expected to help reduce non-radiative recombination in the baseline cell^20,21^. Comparison of the devices indicates that inserting strained wells with thin barriers results in a small improvement in the J_SC_ and negligible degradation in the greater than 1.0 V V_OC_. After adding a two-layer antireflection coating, an encouragingly high one-sun efficiency of greater than 26% in a single-junction quantum well solar cell was obtained under standard AM1.5 illumination.

## Results

**Figure 1:**
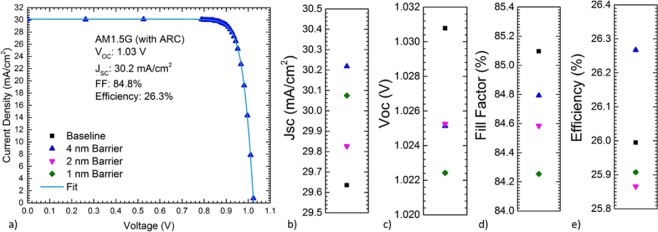
(**a**) AM1.5 G Light I–V and figures of merit (**b**) J_SC_, (**c**) V_OC_, (**d**) fill factor, and (**e**) efficiency comparison between baseline GaAs heterojunction devices with and without strained InGaAs QWs incorporating varying thickness GaAs inter-QW barriers.

Figure 1 displays champion measured 1-sun AM1.5 G light I-V characteristics, representing a remarkable result of a single-junction device, incorporating QWs, with an AM1.5 efficiency over 26%. Figure 1b–e further show comparisons of extracted J_SC_, V_OC_, fill factor, and efficiency of all devices. Device design, fabrication, and testing are described in detail in the Methods section. In all devices, the addition of QWs improved the current density while minimizing loss in V_OC_ to less than 10 mV. Fill factors remained high near 85% and within 1% between device designs. This enabled the 4 nm barrier device to exceed the efficiency of the non-QW baseline.

**Figure 2:**
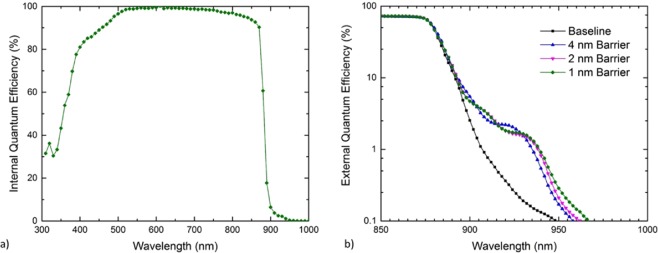
(**a**) Representative internal quantum efficiency of bulk photon collection, as well as (**b**) detail of the sub-bandgap EQE collection through the introduction of InGaAs QWs with varying thickness inter-QW spacers for all heterojunction designs.

The internal quantum efficiency (IQE) of a representative device is shown in Fig. 2a. Modeling of the EQE results using modified drift-diffusion equations indicates that both the emitter and base are of high material quality, specifically with minority carrier diffusion lengths of at least 3x greater than the emitter or base thicknesses, respectively^27^. Figure 2b shows the sub-bandgap external quantum efficiency (EQE) of all devices, where the addition of InGaAs QWs extends the infrared collection relative to the baseline through the introduction of sub-GaAs-bandgap confined states. Moreover, the EQE response in the quantum well samples shift further into the infrared as the barrier thickness decreases. This is evidenced by the increased absorption peaks in the 4 nm barrier sample near 900 nm and 925 nm red-shifting towards 910 nm and 935 nm, respectively, in the thinner barrier samples.

**Figure 3:**
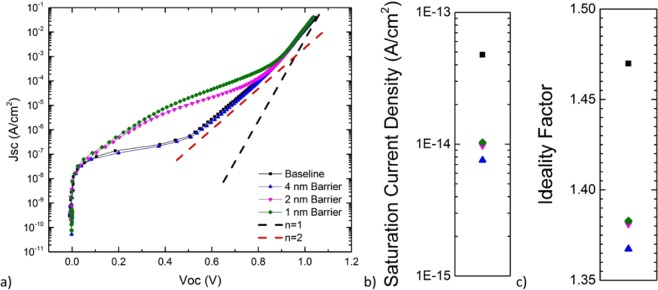
J_sc_-V_oc_ measurement characteristics from all heterojunction devices, including extracted dark saturation current density and ideality factors as fit with a single diode model about the 1-sun V_oc_ point.

A comparison of the diode characteristics, taken using the J_SC_-V_OC_ method to remove the effect of series resistance, of all heterojunction devices are presented in Fig. 3. The saturation current density and ideality factor were extracted by fitting a single diode model against the J_SC_-V_OC_ data near the 1-sun V_OC_ operation point. While a simplification of a more complex system, this allows some first-order comparison of the type and magnitude of recombination mechanisms in these devices. The processes driving the ideality factor towards values of 1 or 2 have sub-components sensitive to recombination in the emitter and recombination in the base. The wider energy gap of the InGaP_2_ emitter should suppress the emitter sub-components, leaving the n = 1 component to be largely driven by carrier diffusion in the base, and the n = 2 to be largely driven by recombination within the portion of the depletion layer residing in the base layer. The dark current and ideality factors were all tightly grouped, and each showed improved characteristics over the baseline device without QWs. This is consistent with a reduction in the relative magnitude of the space charge recombination in this QW device design.

In addition to the n-on-p heterojunction devices presented above, additional samples of p-on-n homojunctions were also fabricated and analyzed. Details of these structures are provided in the Methods section. Although thinning the barrier did not have a strong impact on the standard illuminated IV characteristics in the homojunction structures, analysis of the EL and EQE measurements did reveal a noticeable reduction in the apparent radiative recombination. The 1-sun AM0 characteristics of all devices, without an antireflective coating, are compared in the Supplemental Material (see Figure S1). Similar radiative recombination factors were observed in heterojunction structures, with the added benefit of significantly higher UV collection efficiency, more ideal dark diode characteristics, higher fill factors, and higher overall efficiency. Results pertaining to radiative recombination effects from both sets of devices are presented below.

**Figure 4:**
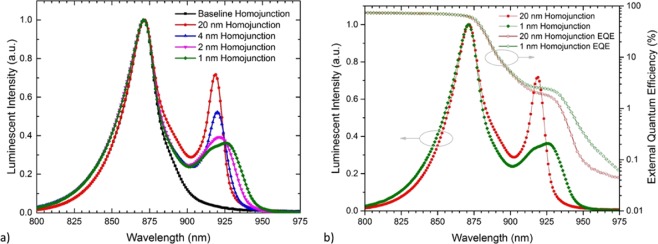
(**a**) Measured normalized EL spectra from GaAs homojunction solar cells incorporating 3-layers of InGaAs QWs with varying barrier thickness, and (**b**) overlay of thick (20 nm) and thin (1 nm) barrier EL data from (**a**) with experimental EQE from the same devices (the full set of EQE spectrum from the homojunction devices are shown in Figure S2 of the Supplemental material).

The effects of reducing barrier thickness on recombination in the QWs is further explained through Fig. 4a, which compares the measured EL spectra of homojunction devices after normalizing to the GaAs peak. As the barrier layer thickness decreases from 20 nm to 1 nm, the dominant InGaAs EL peak red shifts slightly, from 920 nm to 930 nm. In addition, the single peak observed in the 20 nm barrier sample splits into at least two discernable peaks most notably in the 2 and 1 nm barrier samples. This is consistent with bound state splitting as the wells are brought close together. Moreover, the relative intensity of the EL peak from the InGaAs wells to the GaAs bulk also decreases significantly as the barrier layer thickness decreases from 20 nm to 1 nm. Figure 4b compares the thick 20 nm barrier and thin 1 nm barrier EQE directly against the EL, consistently showing the redshift, as well as an increase in carrier collection efficiency, as the barrier is thinned. The decrease in EL intensity, even as the EQE response increases, suggests that the radiative efficiency may be suppressed in the thin barrier structures.

Detailed balance concepts can be used to relate radiative emissions from a semiconductor device to the product of the photovoltaic external quantum efficiency and the equilibrium black body radiation^28^. In general, the radiative luminescence spectrum

1 $${L}_{rad}(E)=EQE(E)\ast BB(E)\ast C$$

is related to the product of the measured EQE spectrum, *EQE*, and the equilibrium blackbody spectrum, *BB* (at a temperature of 25 °C with a cell refractive index of 3.5), each as a function of energy, *E*, with an appropriate scaling factor *C*. In the calculations presented here, the scaling factor in Eq. (1) is assumed to be independent of energy, and the magnitude of the scaling factor is adjusted so that the peak luminescent intensity from the GaAs material (near 875 nm) in the calculated L_rad_ spectrum matches that from the measured EL spectrum.

**Figure 5:**
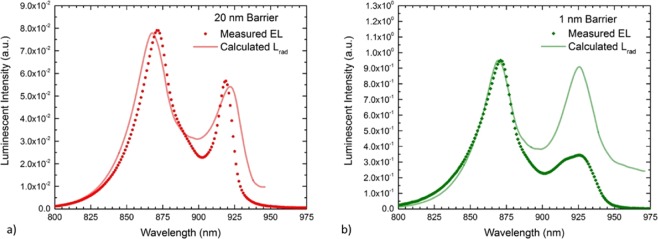
Comparison of the measured luminescence spectra (points) to the calculated L_rad_ spectra inferred from the measured EQE spectra (lines) from GaAs homojunction solar cells incorporating 3-layers of InGaAs QWs with (**a**) 20 nm and (**b**) 1 nm barrier layers.

**Figure 6:**
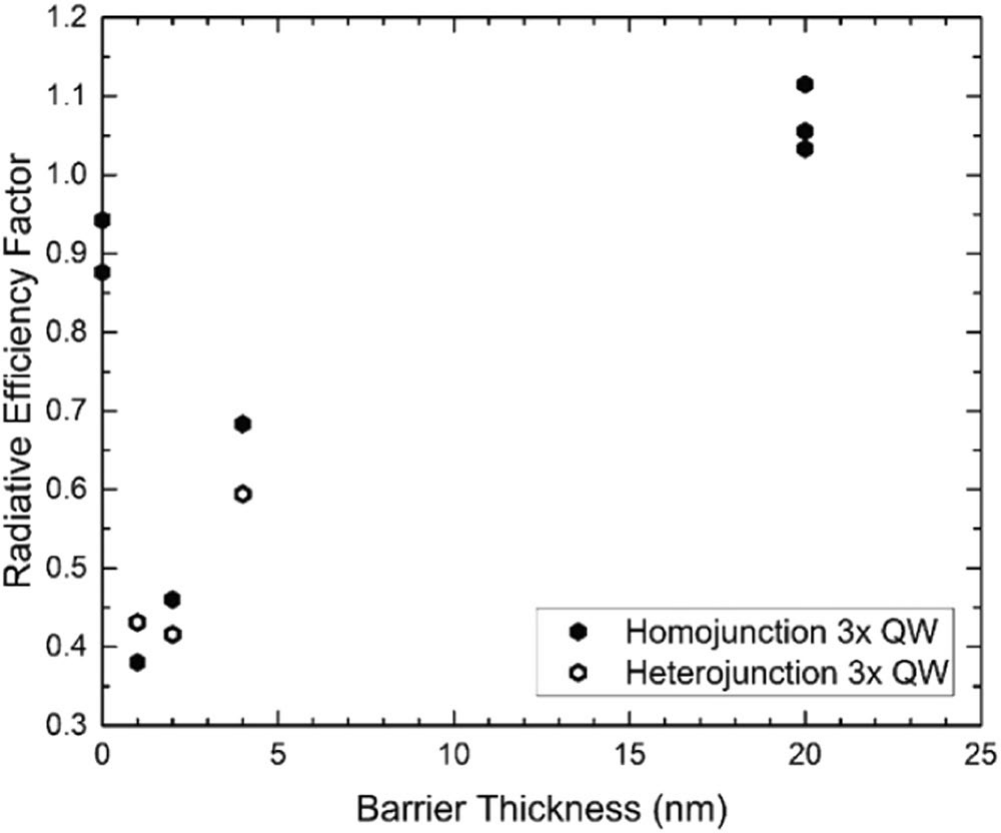
Radiative efficiency factor as a function of barrier thickness for several sets of InGaAs well structures. Solid marks are from P-on-N homojunction cells, while hollow marks are from N-on-P heterojunction cells.

This reciprocity-based analysis concept is demonstrated in Fig. 5, which compares the measured EL spectra to the calculated L_rad_ spectra of the 20 nm and 1 nm barrier samples, respectively. In the 20 nm barrier sample, both the measured EL and extracted L_rad_ spectra show similar QW emission peaks near 920 nm as well as similar relative intensity of GaAs bulk and QW emission. This demonstrates good agreement between the experimental results and the reciprocity relation presented above, and is consistent with radiative recombination in the InGaAs wells behaving similarly to the bulk GaAs radiative recombination rate. While the wavelength of the QW emission for the 1 nm barrier sample was again similar for both the measured EL and extracted L_rad_ spectra, peaking near 930 nm, there was a significant difference in the relative peak intensities. Specifically, the measured EL showed a lower intensity of QW emission compared to the expected result from the reciprocity relation. The lower relative peak intensity is an indicator that the radiative recombination may be suppressed in the InGaAs wells relative to the bulk GaAs radiative recombination rate—an encouraging result towards maximizing device efficiency. To further quantify this analysis, a radiative efficiency factor is defined as the ratio of the relative InGaAs peak intensity from measured EL to the relative InGaAs peak intensity of the L_rad_ calculation. Figure 6 plots this radiative efficiency factor as a function of barrier thickness from both the homojunction and heterojunction devices.

## Discussion

Each of the AR-coated heterojunction devices exhibited 1-sun efficiencies in excess of 25% under 1-sun AM1.5G illumination, as well as fill factors grouped within 1% absolute near 85% which is typical of variation between devices due to the fabrication process. These metrics indicate high quality epitaxial material and fabrication across devices. Short circuit current densities near 30 mA/cm^2^ were realized through the use of a heterojunction InGaP_2_ emitter design and an AlInP front window, significantly reducing parasitic absorption from the InGaP_2_ window of GaAs homojunction designs (as seen in the Supplemental Material Figure S2a). The V_OC_ of the baseline device was typical for a high-quality single-junction GaAs device without incorporating a back reflector or photon recycling. The devices incorporating QWs exhibited reduced V_OC_ from the baseline, which has often been seen with such designs. Significantly, however, the V_OC_ of the nanostructured devices was maintained above 1.0 V and indeed the voltage loss was less than 10 mV as compared to the baseline. The sub-bandgap EQE shows a redshift with decreasing barrier width, which is consistent with state splitting caused by the proximity of the three wells and was further confirmed through simulation of the system by 8-band k∙p calculations in the commercial software package nextnano. Integrating the sub-bandgap EQE from 880 nm to 1000 nm against AM1.5 G yields 0.194 mA/cm^2^ for the baseline and 0.252 mA/cm^2^ to 0.258 mA/cm^2^ for the QW devices, which corresponds to approximately 19.1 μA/cm^2^ collection from each individual QW layer. This is consistent with previous reports of current collection per layer of more complicated InAs quantum dot structures^29^.

Some variation was observed in J_SC_-V_OC_ results below ~0.9 V where a voltage dependent recombination mechanism exists in the 2- and 1-nm devices, but does not appear in the 4-nm and baseline devices. This may be caused by edge recombination effects or non-ideal fabrication, or it may be caused by other more complicated phenomena such as tunneling-assisted recombination at preferential voltage alignments of bound states as the barrier thins^30^. While this variability would impact devices operating in this part of the diode curve the data converges near ~0.9 V and beyond and does not impact performance under 1-sun or low-concentration illumination. The dark current and ideality factors show the highest recombination rate, and highest ideality factor, in the baseline device, with improved (lower) results from the QW devices. Results from the homojunction devices are shown in Figure S3, where the baseline device has the lowest dark current and ideality factor, though these values decrease as the barrier is lowered from 4 nm to 0 nm, which does not follow the same trend as the V_OC_ shown in Figure S1c. As V_OC_ is inversely dependent on dark current magnitude, the fact that the baseline V_OC_ remains higher than the QW devices suggests a more complicated relationship.

To better understand the impact of the structure design that combines a strained quantum well superlattice with a heterojunction emitter, the n = 1 and n = 2 saturation current densities were extracted from both the measured QW and baseline cells. Using a simple model described further in the Methods section, a radiative recombination coefficient and a non-radiative space charge recombination coefficient (B_rad,2D_ and A_SCR,2D_, respectively) is quantified and compared against earlier reports on stain-balanced quantum well devices^18,19^.

Both non-radiative and radiative processes contribute to the dark current of III–V semiconductor diodes, and these process can have different voltage dependences. Radiative processes have an n = 1 voltage dependence, and in the best cases, will ultimately limit the voltage performance of photovoltaic devices as discussed in the previous section. However, in practice, non-radiative recombination often limits the diode dark current. Non-radiative dark current processes can follow either an n = 1 or n = 2 voltage dependence, depending upon the location of the recombination within the device. Non-radiative events in the quasi-neutral regions of the device contribute to the n = 1 component, while non-radiative events within the junction depletion region contribute to the n = 2 space charge recombination component of the diode dark current. Here, the dark diode current of the baseline cell is assumed to be the sum of n = 1 and n = 2 components, with the n = 1 component being a mixture of both radiative and non-radiative recombination. Since the QWs reside within the junction depletion region, the non-radiative recombination in the wells is assumed to add incrementally only to the n = 2 component. On the other hand, radiative recombination from the wells is assumed to add incrementally to the n = 1 component of the diode current.

Table 1 compares the extracted dark current parameters from this work on strained QW solar cells (heterojunction devices) to several prior reports on strain-balanced QW solar cells. The n = 1 saturation current density in the baseline cell (${J}_{01}^{B}$) is over an order of magnitude lower in this work relative to some prior reports, while the n = 2 saturation current density in the baseline cell (${J}_{02}^{B}$) is nearly 3x lower. This reduction in dark current of the baseline cell can be attributed, at least in part, to the use of a heterojunction emitter and the subsequent reduction of both n = 1 and n = 2 non-radiative components^20^.

**Table 1:** Dark diode parameters extracted from measured current-voltage characteristics in this work and in several prior reports^18,19^.

Device Structure	Baseline Cell		Quantum Structured Absorber		
	${{\boldsymbol{J}}}_{01}^{{\boldsymbol{B}}}$ (mA/cm^2^)	${{\boldsymbol{J}}}_{02}^{{\boldsymbol{B}}}$ (mA/cm^2^)	λ_edge_ (nm)	B_rad,2D_ (μm^2^/s)	A_SCR,2D_ (ns^−1^)
Ref.^18^ (strain balance)	1.0 × 10^−15^	2.0 × 10^−8^	930	1.0 × 10^5^	2.9
Ref.^19^ (strain balance)	2.5 × 10^−16^	1.5 × 10^−8^	925	2.5 × 10^5^	2.25
This work (strained)	1.0 × 10^−16^	6.5 × 10^−9^	940	1.0 × 10^4^	0.278

In addition to the improved baseline cell, both the radiative recombination coefficient, ${B}_{rad,2D}$, and non-radiative space charge recombination coefficient, ${A}_{SCR,2D}$, describing the impact of the quantum structured absorber region on the diode current are roughly an order of magnitude lower in this work. This reduction in recombination in the quantum wells is attributed, at least in part, to the use of a strained multiple quantum well structure with thin barriers that exhibits the reduced carrier overlap characteristic of a superlattice under an electric field. The presence of an electric field is an important consideration, as the quantum well region in this work (and many others) is placed within the depletion region of a p-i-n junction, and thus is subject to a built-in electric field. Prior work has discussed in detail the impact of field strength on carrier collection efficiency and radiative lifetime in quantum structured solar cells^29^.

In brief, when a quantum well is subjected to an electric field, electrons are pushed toward one side of the well and holes to the other, causing a reduction in the wavefunction overlap, typically referred to as the quantum-confined Stark effect (QCSE)^30^. The QCSE in quantum well structures becomes more pronounced as the field strength and/or well thickness increases. In multiple quantum well structures, an additional separation of the carrier wavefunctions can occur as the barrier thickness between the wells is reduced and the wavefunctions begin to couple between the wells. As previously discussed, the observed redshift in the EQE and EL spectrum with decreasing barrier thickness is consistent with increased wavefunction coupling. The behavior of the radiative efficiency factor is also consistent with these expected changes in carrier overlap. As seen earlier in Fig. 6, the highest inferred radiative efficiency factor was in the multiple quantum well structures with thick barriers (>4 nm) and no expected coupling between the wells. A slightly lower radiative efficiency factor is observed in the structure with no barriers, e.g. a thicker InGaAs well structure (27.6 nm vs. 9.2 nm). The use of thinner quantum wells is expected to force a tighter overlap of the electron and hole wave functions, and thus enhance luminescence, as observed. On the other hand, the lowest radiative efficiency factor is obtained in thin barrier (<4 nm) structures in which coupling between the wells is expected. Carrier transport through the resulting superlattice can further separate carriers and suppress radiative recombination, consistent with the observed reduction in radiative efficiency factor.

It has previously been suggested that if carrier transport through a superlattice is efficient, electrons and holes may accumulate on opposite ends of a quantum well superlattice^31^. In addition to the reduction in radiative recombination suggested above, such a separation in carriers is expected to have an impact on both non-radiative recombination and carrier escape mechanisms. While advanced device concepts such as two-step photon absorption may benefit from enhanced carrier separation, thermal escape has been previously shown to be the dominant escape mechanism in GaAs-based quantum structures at room temperature and moderate fields^25,29^. The observed enhancement in carrier collection in thin-barrier structures observed in this work is thus the likely result of the reduction in recombination followed by efficient thermal escape from both ends of the superlattice.

Finally, we suggest that a reduction in carrier overlap in strained superlattice structures may have one additional impact of significant note for photovoltaic applications, namely an increased non-isotropic emission pattern. While not yet measured in the devices reported here, prior work on strained quantum well solar cells and LEDs has demonstrated polarized light emissions and an effective restriction in angular luminescent emissions from the quantum well region^15,32^. Restricting the angular emissions of the absorber region of a photovoltaic device can in principle reduce overall radiative emissions, and thus increase the limiting efficiency performance^33^. Earlier detailed balance calculations suggest that restricted radiative emissions can have a significant impact on the open circuit voltage and efficiency of lower energy-gap cells operating in the radiative limit^34,35^. A strained quantum well superlattice solar cell such as the one described in this work may thus represent a practical realization of a restricted emission photovoltaic device with a limiting efficiency in excess of 40% in a single-junction structure.

## Conclusions

Nanostructured quantum well and quantum dot solar cells have been widely investigated as a means of extending infrared absorption and enhancing III–V photovoltaic device performance. While nanostructured III–V absorbers have been successfully used to improve current matching in multijunction devices, successful demonstrations of high-performance single-junction devices have, in the past, proven more elusive. In this work, a single-junction nanostructured III–V solar cell with 1-sun efficiency exceeding 26% has been demonstrated under AM1.5 illumination. This high efficiency has been obtained by combining a low dark current heterojunction with a strained quantum well superlattice structure that exhibits signs of suppressed radiative recombination and enhanced carrier collection. A study of the impact of barrier thickness on infrared external quantum efficiency and relative luminescence intensity suggests enhanced carrier separation within the quantum well region can play a key role in enhancing the performance of multiple quantum well solar cells with thin barriers (<4 nm). Further improvements in device performance are anticipated with the addition of light-trapping structures and/or partial strain-compensation techniques, as strained quantum well superlattice solar cells may represent a practical realization of restricted emission photovoltaic devices.

## Methods

All devices were grown on n- or p- doped 2″ GaAs (100) 2° <110> substrates using a 3 × 2″ Aixtron close-coupled showerhead metal organic vapor phase epitaxy (CCS-MOVPE) reactor. Standard group III precursors of trimethylindium, trimethylgallium, and trimethylaluminum were used, along with arsine and phosphine group V sources. Diethylzinc was used as the p-type dopant source and disilane along with diethyltellurium were used as the n-type dopants. Materials were grown at 650 °C (substrate surface temperature as measured with an *in situ* Laytec pyrometer system). V/III ratios used were typically between 50–200 depending on material. Growth rates for all materials were between 1 and 2 μm/hr.

### P-on-N Homojunction Structure

**Figure 7:**
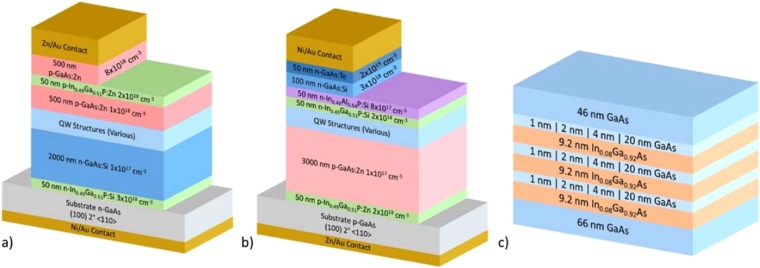
Epitaxial layer structure of the (**a**) P-on-N homojunction and (**b**) N-on-P heterojunction devices with variable QW structures (**c**) including either 1, 2, 4, or 20 nm GaAs barriers between InGaAs QWs.

The p-i-n GaAs homojunction design, including film thicknesses, compositions, and doping levels, is shown in Fig. 7a. Six iterations of the QW region were used, four of which are detailed in Fig. 7c: a 3-layer In_0.08_Ga_0.92_As/GaAs superlattice in the i-region with different GaAs barrier thicknesses varying between 1 nm and 20 nm. A fifth iteration replaced the 3-layer QW/barrier regions with a single 27.6 nm thick In_0.08_Ga_0.92_As well, essentially a 3x QW structure with 0 nm thick barriers. Finally, the sixth iteration was a baseline device with the “QW Structures” layer replaced with 100 nm of GaAs. No intentional doping was used for any of the materials in the variable QW regions of these six iterations. The critical thickness for the strained QWs was determined to be approximately 30 nm through investigation of photoluminescence intensity and reciprocal space mapping as a function of InGaAs thickness (not shown). Hence, the well thickness for the 3-layer superlattice was kept constant at 9.2 nm to keep the total InGaAs thickness below the critical thickness.

### N-on-P heterojunction structure

The n-i-p InGaP-GaAs heterojunction design, including film thicknesses, compositions, and doping levels, is shown in Fig. 7b. Four iterations of the QW design were used in this structure: a baseline with no QWs in the i-region and a QW device with a 3-layer In_0.08_Ga_0.92_As/GaAs superlattice in the i-region with three different GaAs barrier thicknesses: 1 nm, 2 nm, and 4 nm, as shown in Figure c but without the 20 nm barrier design.

### Device fabrication and testing

Twelve 1 × 1 cm^2^ cells were fabricated on each 2″ wafer using conventional III/V processing techniques. The best representative cell from each wafer was used to characterize each of the six p-on-n homojunction structures and each of the four n-on-p heterojunction structures. Contact lithography was used to define the front contact metal grid regions. These were electroplated with 5 nm Ni and 1.6 μm Au for n-type ohmic contacts, or 5 nm Zn and 2 μm Au for p-type ohmic contacts, using commercial plating solutions purchased from Transene. Contact lithography was then used to define the active areas which were isolated using a wet chemical etch. Transmission line measurement test structures were used to confirm ohmic contacts on all samples with contact resistance of 3 × 10^−5^ Ω-cm^2^ or better. The heterojunction samples were then coated with an anti-reflection coating (ARC) comprised of 55 nm ZnS and 85 nm MgF_2_, which was optimized to minimize surface reflection loss from 300 to 1000 nm.

SR and EQE measurements were taken using a Newport IQE 200 quantum efficiency measurement system which was calibrated using both Newport silicon (Model 818-UV-L) and Newport germanium (Model 818-IR-L) reference detectors. 1-sun AM1.5G and AM0 illuminated I-V measurements were performed using a dual source 18 kW solar simulator system custom built by TS Space Systems. The system was calibrated using InGaP_2_ (Eg = 1.85 eV) and GaAs (Eg = 1.42 eV) cells calibrated by the National Renewable Energy Laboratory (NREL). The spectral match between RIT AM1.5 G/AM0 and ASTM AM1.5 G/AM0 is nearly unity from 350 nm to 1200 nm with exception of out of range points between 600 nm–700 nm. During the measurement, the samples were placed on a temperature-controlled chuck set at 25 °C. Both the dark and illuminated diode behavior was measured using a Keithley 2400 source meter. Electroluminescence was taken by closely coupling the fiber optic of an ASD FieldSpec 3 spectroradiometer with the solar cell under an injection current of 100 mA/cm² with an integration time of 68 ms.

### Practical device model for nanostructured solar cells

A simple practical device model is used to characterize nanostructured solar cell performance by assuming the incorporation of quantum wells (or dots) contributes to both the n = 1 and n = 2 component of the dark diode current as well as the infrared collection, and that these contributions scale with the number of quantum well (or dot) layers. Here, n is the ideality factor described in the Shockley ideal diode equation. Because the wells typically reside within the junction space charge region (SCR), non-radiative recombination is expected to contribute only to the n = 2 space charge recombination component. In contrast, radiative recombination is assumed to contribute to the n = 1 component. The extended infrared absorption is characterized in terms of the fraction of the light absorbed per layer above the effective absorption edge of the nanostructured region (λ_edge_).

Space charge recombination is well known to depend upon the trap density (N_t_) within the depletion region, the intrinsic carrier concentration (n_i_) and the width of the space charge recombination region. The intrinsic carrier concentration is, in turn, strongly dependent upon the energy-gap (E_g_), decreasing rapidly with increasing subcell energy-gap. In this work, it is assumed that the nanostructured layers are of fixed energy gap and are placed within the space charge region, such that the n = 2 saturation current density (${J}_{02}$) scales with layer number (${M}_{QW}$):

2 $${J}_{02}={J}_{02}^{B}+q{N}_{2D}{e}^{(-{E}_{g}/2kT)}{A}_{SCR,2D}{M}_{QW}$$

where ${J}_{02}^{B}$ is the n = 2 component of the baseline cell, ${A}_{SCR,2D}$ is the non-radiative space charge recombination coefficient, and *E*_*g*_ is defined by the absorption edge of the nanostructured layers. Here we assume that carrier density in each layer can be characterized by a fixed two-dimensional carrier concentration ${N}_{2D}$ = 1 × 10^12^ cm^−2^ that increases exponentially with decreasing energy gap, consistent with previous work^21^. We note that minimizing ${A}_{SCR,2D}\,$will depend on reducing the defect density in the nanostructured absorber, as well as optimizing the position of the wells (or dots) within the depletion region.

Radiative recombination in each nanostructured layer is described mechanistically in terms of carrier recombination via the use of a two-dimensional radiative recombination coefficient (${B}_{rad,2D}$)^36^. Specifically, radiative recombination in the quantum well (or dot) layers is proportional to the product of the two-dimensional radiative recombination coefficient and the carrier densities. Here we assume that each (electron and hole) carrier density can be characterized by a fixed two-dimensional carrier concentration in each layer that increases exponentially with decreasing energy gap, as detailed above. Further assuming that the radiative recombination increases linearly with the number of layers (e.g. the layers emit evenly), the radiative saturated current density (${J}_{01}$) generated by multiple layer structures can be expressed as:

3 $${J}_{01}={J}_{01}^{B}+q{{N}_{2D}}^{2}{e}^{(-{E}_{g}/kT)}{B}_{rad,2D}{M}_{QW}$$

where ${J}_{01}^{B}$ is the n = 1 component of the baseline cell and ${M}_{QW}$ is again the number of quantum well or quantum dot layers. In practice, we note that the addition of wells (or dots) could generate defects that propagate outside the junction depletion region and increase the non-radiative recombination in the baseline cell. In the simple model summarized by Eqs (2) and (3), this situation would result in an apparent increase in ${B}_{rad,2D}$ as well as ${A}_{SCR,2D}$.

In Table 1, the baseline cell parameters were extracted from fits of the illuminated-current voltage characteristics of the baseline cell without quantum wells, assuming the measured short circuit current density, a variable series resistance, and a standard two-diode model of the dark current. The n = 1 and n = 2 saturation current density of the quantum well devices were then obtained from a similar fit of the illuminated-current voltage characteristics. Such a fit is plotted in Fig. 1 along with the measured data from the 26.3% efficient quantum well solar cell device. The quantum structured absorber parameters listed in Table 1 were then derived from these fitted parameters using Eqs (2) and (3).

## Supplementary Information

Supplementary Information

## Data Availability

The data described in this study (including Supplementary Information) are available from the corresponding author upon request.
