# Supplementary material for: Design and Demonstration of High-Efficiency Quantum Well Solar Cells Employing Thin Strained Superlattices
Source: Sci Rep. 2019 Sep 27;9:13955. doi: 10.1038/s41598-019-50321-x (PMC6765021; doi:10.1038/s41598-019-50321-x)
Supplement: Supplementary file 1 — Supplementary Information [file 41598_2019_50321_MOESM1_ESM.docx]

**Supplementary Information**

**Design and Demonstration of High-Efficiency Quantum Well Solar Cells Employing Thin Strained Superlattices**

**Roger E. Welser,^1^ Stephen J. Polly,^2^ Mitsul Kacharia,^2^**

**Anastasiia Fedorenko^2^, Ashok K. Sood,^1^ and Seth M. Hubbard^2^**

^1^ Magnolia Optical Technologies, Inc. 52-B Cummings Park, Suite 314, Woburn, MA 01801

^2^ NanoPower Research Laboratories, Rochester Institute of Technology, Rochester, NY 14623


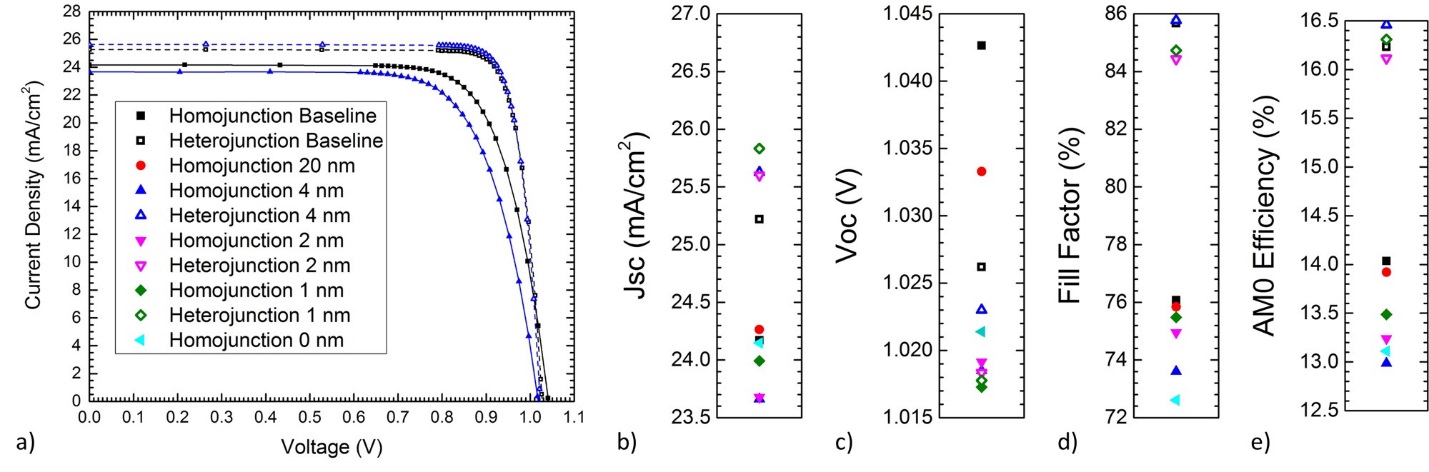


Figure S1: Comparison of 1-sun AM0 experimental results for all devices. a) compares the baseline and 4 nm barrier samples of the homojunction (solid markers) and heterojunction (hollow markers), while b), c), d), and e) compare the J_SC_, V_OC_, Fill Factor and AM0 efficiency, respectively, of all devices. The heterojunctions exhibited higher J_SC_ due to improved quantum efficiency over homojunction designs (see Figure S2a). The homojunction control had an improved V_OC_ over the heterojunction, possibly due to differences in doping level and quasi-Fermi level split between the two device designs. Lower fill factor in the homojunction devices was likely due to fabrication of thinner front contact metallization than designed for.


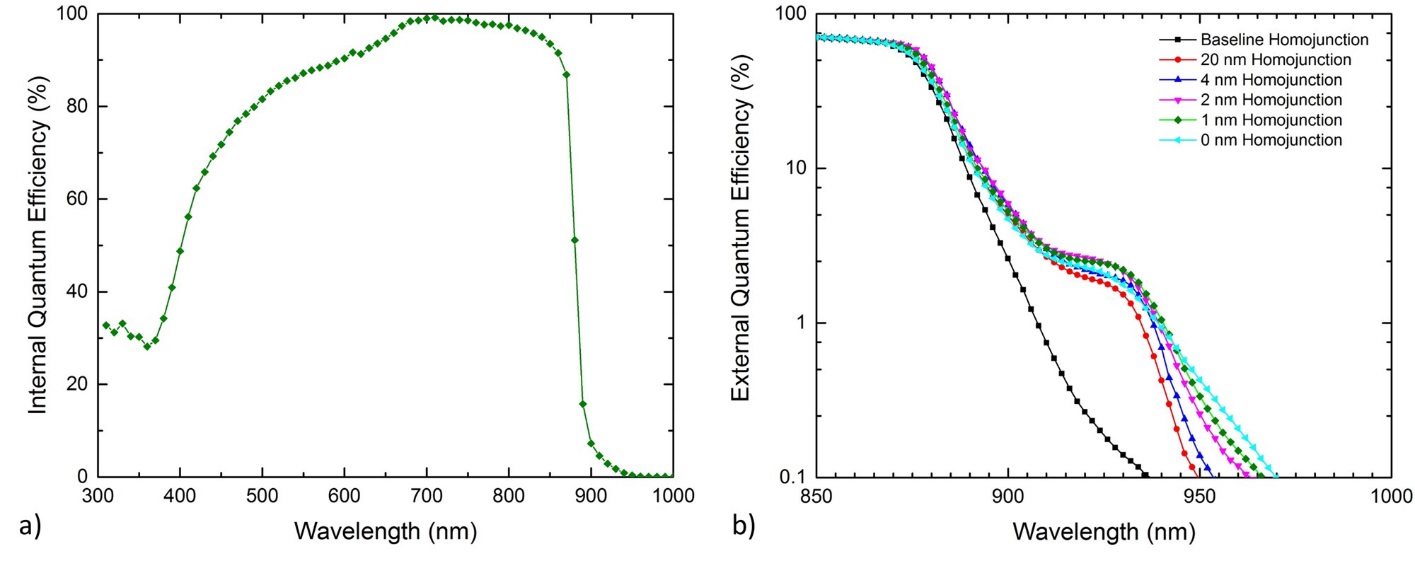


Figure S2: a) Internal quantum efficiency of the homojunction device design, showing parasitic absorption from the InGaP2 front window (~400 nm to ~670 nm) as compared to the heterojunction shown in Figure 2a. b) sub-bandgap EQE of all homojunction devices, exhibiting a similar redshift observed by the heterojunction designs as the QW barrier was thinned.


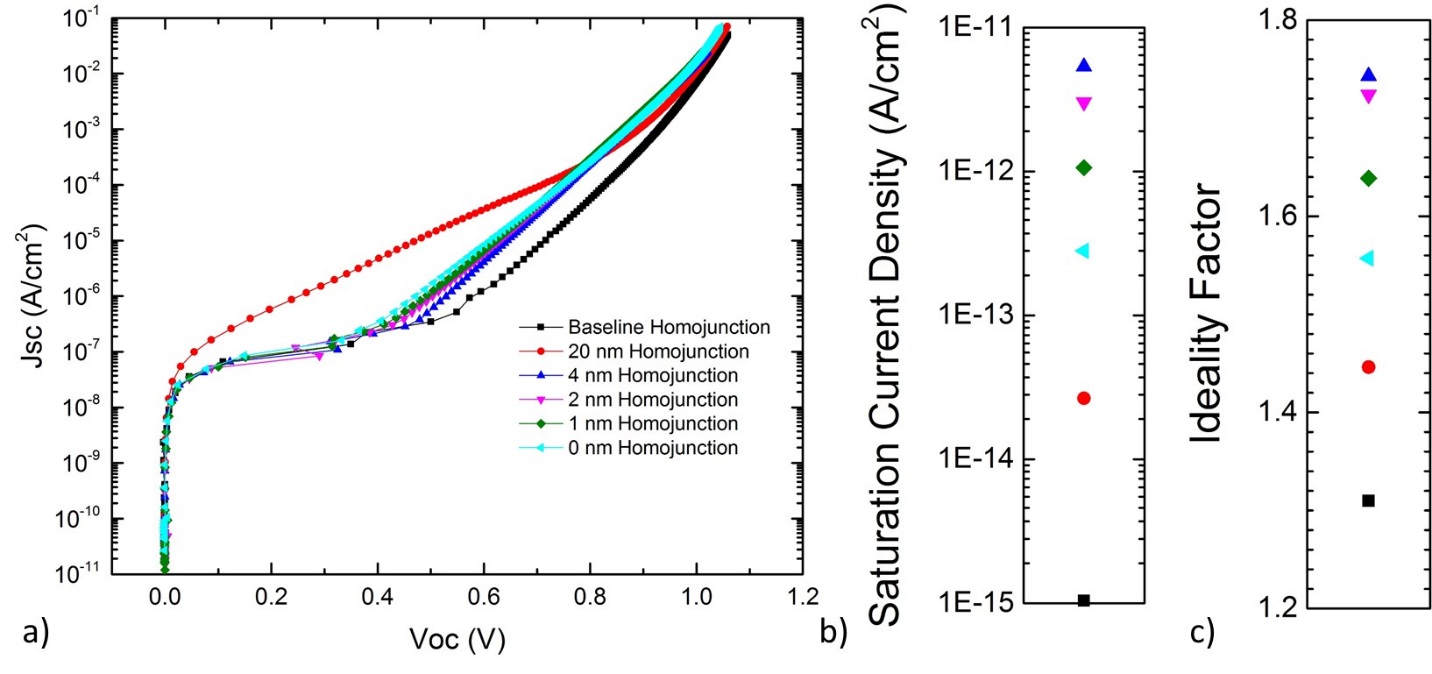


Figure S3: a) J_sc_-V_oc_ measurement characteristics from all homojunction devices, including extracted dark saturation current density and ideality factors as fit with a single diode model about the 1-sun V_oc_ point.
